# The effect of diurnal variation on the performance of exhaustive continuous and alternated-intensity cycling exercises

**DOI:** 10.1371/journal.pone.0244191

**Published:** 2020-12-31

**Authors:** Amine Souissi, Narimen Yousfi, Nizar Souissi, Monoem Haddad, Tarak Driss

**Affiliations:** 1 Interdisciplinary Laboratory in Neurosciences, Physiology and Psychology, Physical Activity, Health and Learning (LINP2-2APS), UFR STAPS, UPL, Paris Nanterre University, Nanterre, France; 2 Physical Activity, Sport and Health, UR18JS01, National Observatory of Sport, Tunis, Tunisia; 3 Research Laboratory “Sport Performance Optimization”, National Centre of Medicine and Science in Sport (CNMSS), Tunis, Tunisia; 4 Physical Education Department, College of Education, Qatar University, Doha, Qatar; University of Bourgogne France Comté, FRANCE

## Abstract

The purpose of this study was to explore the effect of time of day (TD) on two types of exercise protocols [continuous (CP) *versus* alternated (AP)]. Eleven physical education students (mean ± SD: age = 24.4 ± 1.2 years, aerobic peak power (APP) = 290 ± 31.9 W) underwent four sessions. These sessions were performed at 08:00 (AM) and 18:00 (PM) and consisted of cycling exercises until voluntary exhaustion at 90% APP (CP) or 70%-105% APP (AP) with the order of testing randomly assigned. Time to exhaustion (time limit = Tlim) was measured from the start of the test to when voluntary exhaustion occurred. Heart rate (HR) was recorded at baseline (HRbaseline) and throughout the protocols to determine HR at exhaustion (HRpeak). Blood lactate ([La]) was measured at rest, immediately after exhaustion and at 2min30 post-exercise to determine [La]peak. A significantly higher means of Tlim (334 ± 57 s; 272 ± 59 s; p< 0.05), HRbaseline (72 ± 5 beats/min; 67 ± 5 beats/min; p< 0.01), HRpeak (186 ± 8 beats/min; 178 ± 9 beats/min; p< 0.01) and [La]peak (16.2 ± 2.1 mmol/l; 13.9 ± 1.9 mmol/l; p< 0.05) were observed in CP at the PM compared to the AM. In addition, a significant higher means of Tlim (380 ± 54 s; 312 ± 82 s; p< 0.05), HRbaseline (73.1 ± 5.5 beats/min; 67 ± 5.4 beats/min; p< 0.01), HRpeak (186 ± 8 beats/min; 180 ± 9 beats/min; p< 0.05) and [La]peak (17.9 ± 1.8 mmol/l; 14.7 ± 2.1 mmol/l; p< 0.01) were observed in AP at the PM compared to the AM. It is concluded that AP and CP are more appropriate in the late afternoon than in the morning for performing long-lasting exercises. The AP could be a novel strategy for increasing the engagement in physical activity.

## Introduction

Many physiological circadian rhythms at rest are endogenously controlled, and persist when an individual is isolated from environmental fluctuations [1–6]. The circadian rhythm of resting core body temperature (BT) is well-documented [7–11]. The BT decreases to a minimum during sleep from 2:00 AM to 6:00 AM and begins to rise before wakefulness until the acrophase of the rhythm is reached at around 6:00 PM [12, 13], as well as the circadian variation of heart rate (HR) [14, 15], both, demonstrating concomitant patterns throughout 24 hours [16]. Indeed, BT shows a robust circadian rhythm with an endogenous component that is at least as large as the exogenous component due to the sleep–wake cycle [5, 6]. In fact, a robust circadian variation in vascular function occurs in healthy humans and exhibits an unfavorable profile in the morning hours [17]. A reduced function of the intrinsic endothelial nitric oxide-vasodilator system was observed in the morning [17, 18]. In addition, higher concentrations of vasodilating factors such as histamine [19] in the early evening may favor greater vasodilation when exercise is performed at this time of day (TD).

Many chronobiological field studies have demonstrated diurnal variation in cycling performance [1, 20–26]. Poorer performances are commonly observed in the AM, while best performances are attained in the PM [5, 25–27]. Several authors have claimed that the daily variations in short-term performance are paralleled closely with that of BT [28–30] but the exact mechanism(s) for a causal link between BT and human performance are still unclear and require more research [31]. The existences of TD effects on short-term exercise (≤ 6 sec) involving anaerobic metabolism have previously been established [25, 28, 32]. However, few studies have investigated the TD effect on aerobic performance. A study conducted by Souissi et al. (2007) used the Wingate test to explore the effects of TD on the aerobic and anaerobic contribution to performance found that oxygen uptake increased significantly during the Wingate test from morning to afternoon, which may enhance aerobic contribution to performance in the afternoon [33]. Furthermore, Fernandes et al. (2014) [34] showed that oxygen uptake and aerobic mechanical power output at 600 and 1000 m tended to be higher in the evening. The aerobic contribution was estimated to be 16% during the Wingate test [35] and 79% during ≈4min of exhaustive exercise [36]. The aerobic system plays a major role in determining performance over long duration (greater than 4min) of continuous and severe-intensity intermittent exercise [37]. It will be interesting therefore to investigate the TD effect on the performance using exhaustive cycling exercises eliciting 90% of maximal aerobic power to evaluate the TD effect on aerobic performance.

Zinoubi et al. (2018) [38] showed that HR values, lactate ([La]) and rating of perceived exertion (RPE) scores measured during constant-intensity exercises were higher than those measured during alternated-intensity exercise with the same average power output. However, since decreasing power output increased aerobic contribution during intermittent exercise [37] and the aerobic contribution was higher at the PM compared to the AM during high-intensity cycling exercise [21], one could speculate that time to voluntary exhaustion (time limit = Tlim) will be greater in the continuous protocol (CP) than alternated protocol (AP), but the difference of Tlim between protocols could be attenuated at the PM. To the best of our knowledge, no study has compared the Tlim of CP with AP (with the same average output) in cycling exercise. Furthermore, the effect of TD on HR, [La] and Tlim in alternated-intensity cycling exercises with external work varying according to a defined pattern (i.e., AP) is not yet studied. Thus, the purpose of this study was to investigate the time of day effect on time to voluntary exhaustion and physiological responses to alternated protocol and continuous protocol.

## Materials and methods

### Participants

Eleven healthy male, physical education students (mean ± SD: age = 24.4 ± 1.2 years; body mass = 81.4 ± 13.2 kg; height = 1.8 ± 0.06 m) participated in the present study. The aerobic peak power (APP) of the participants was 290 ± 31.9 W (range: 240 W—320 W). They were selected based on their chronotype using the questionnaire by Horne and Ostberg (Horne and Ostberg 1976). All participants had an intermediate chronotype profile (range: 47–55, mean ± SD: 51 ± 2.6), they were amateur soccer players, nonsmokers, and they refrained from exercise and alcohol- and caffeine-containing drinks for at least 24 h before the measurements started. They were instructed to maintain the same dietary patterns during the 24 hours before each experimental condition. Each participant received thorough explanations about the protocol and signed a written informed consent form prior to participation. The experimental protocol was approved by the Institutional Review Board of Nanterre University, and carried out according to the guidelines of the Declaration of Helsinki.

### Experimental design

Before beginning the study, the participants were familiarized with incremental, constant and alternated-intensity exercises by performing short bouts of each protocol on a mechanically braked cycle ergometer (Monark 894E, Varberg, Sweden). During the first session after the familiarization, an incremental cycling test to exhaustion as described by Zinoubi et al. (2018) [38] was performed to estimate the aerobic peak power (APP) of each participant. The participants performed an incremental cycling test until exhaustion at a constant pedal rate of 80 RPM (ranging from 79 to 82) to determine the aerobic peak power (APP). This test began at 80 W. The power output was increased by 20 W every minute. The power output corresponding to the last step of this incremental test was considered as APP. Following the incremental cycling test, each participant completed a cycling exercise at constant or alternated intensities, in the morning 08:00 (AM) or in the late afternoon 18:00 (PM). The sessions were randomized and counterbalanced in order of administration to minimize any learning effects [11, 25]. The intervals between testing sessions for recovery were at least equal to 48 hours. The mean ambient temperature and relative humidity of the laboratory were kept stable (22 ± 0.1°C and 40 ± 0.4%, respectively).

### Protocol

**Fig 1:**
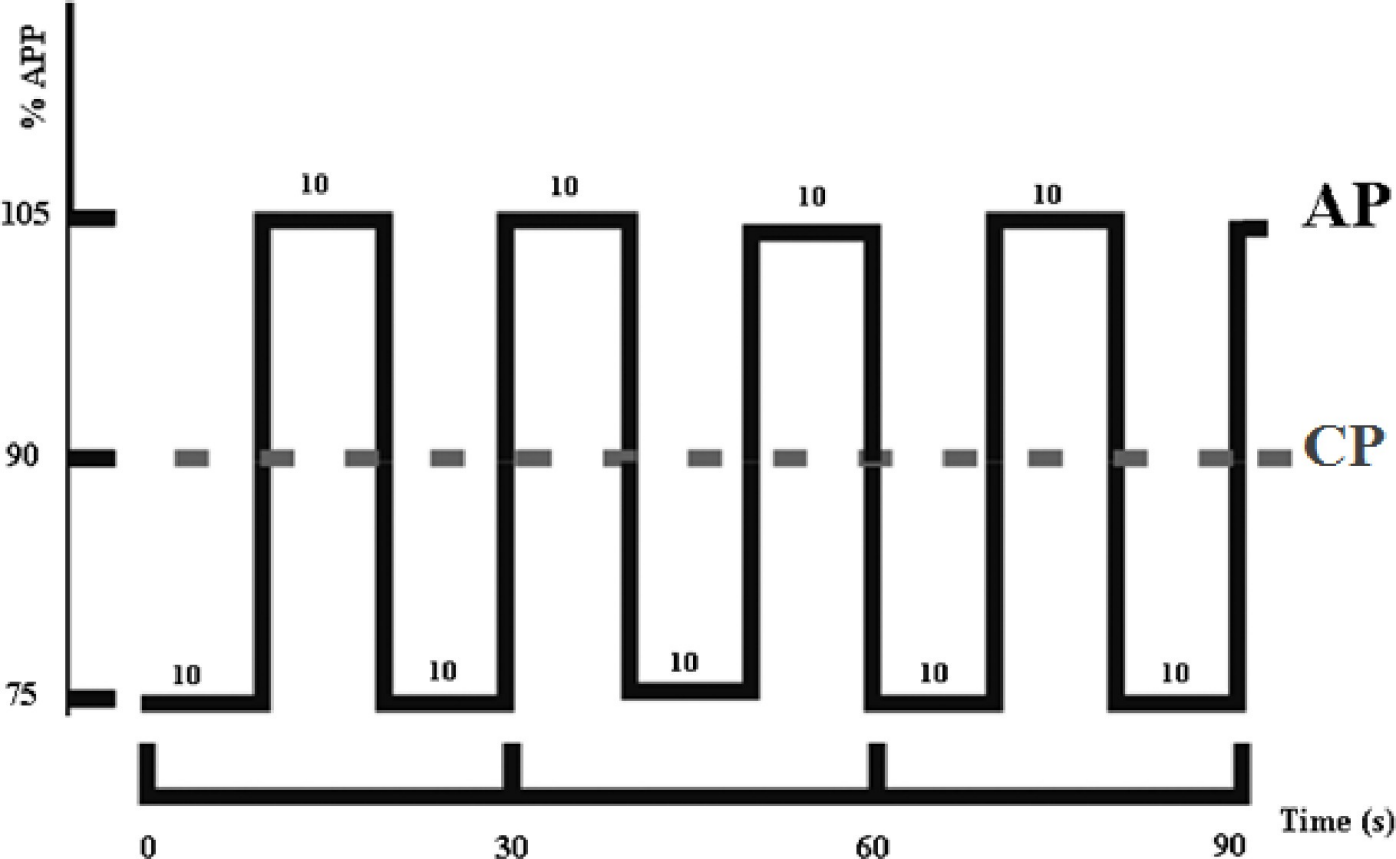
Experimental protocol; CP: Continuous protocol; AP: Alternated protocol.

The participants were instructed to eat the same breakfast (approximately at 06:30) and were strictly supervised by one experimenter. In the morning, participants arrived at the laboratory at 07:30 when they were requested to wear the HR belt and to get ready for the test. Before the late afternoon session, the last meal was consumed at the most 5 hours before the test (supervised by one experimenter). In the afternoon, participants arrived at the laboratory at 17:30. Each test started with a warm-up cycling exercise of 5 minutes at 40% APP and a passive recovery (until HR decreased to <110 bpm). During the test the seat height was adjusted and kept the same for each participant in all trials. The feet were fixed to the pedals with toe-clips. Following the warm-up, the participants were instructed to pedal a cycle ergometer in a sitting position during CP or AP and were encouraged and checked to maintain a pedaling frequency of 80 RPM at 90% APP (on average) until voluntary exhaustion. During the AP, the intensity changed every 10 s (75% APP and 105% APP). Alternated intensities were set by adding or subtracting APP *0.3/80 RPM (30% APP) (Fig 1). The participants started cycling at 75% APP. The HR was recorded continuously with a Sport-Tester (Polar RS400, Finland) to determine the HR at exhaustion (HRpeak). [La] capillary blood samples (5 μl) were collected from the fingertip for lactate concentration (mmol · l-1) at rest, immediately after exercise and at 2min30 post-exercise to determine [La]peak using a portable blood lactate analyser (Lactate Pro, Japan). The lactate analyser was calibrated before each test and was used according to the manufacturer’s guidelines. The RPE was also recorded immediately after the end of the exercise using a French translated [39] Borg 6–20 point-category scale [40].

### Statistical analyses

Statistica software (Statsoft, Maison Alfort, France) was used for all analyses. The data were analyzed by means of Statistica software (Statsoft, Maison Alfort, France), using a General Linear Model with a two way ANOVA with repeated measures (type of exercise × TD). The Bonferroni test was used to identify significant differences. Effect sizes were calculated as partial eta-squared (η_p_^2^) to assess the practical significance of our findings. Magnitudes of effect sizes were classified as trivial (0–0.19), small (0.20–0.49), medium (0.50–0.79), and large (≥0.80) [41]. The level of significance was predetermined to be P < 0.05 for all statistical analyses. The results are presented as the mean ± SD throughout the text.

## Results

Mean ± SD values for HR, [La], Tlim and RPE are presented in Table 1. No significant (TD × protocol) interaction was obtained for Tlim, HR, [La] and RPE (P > 0.05).

**Table 1:** Values (mean ± SD) of Tlim, HR, [La] and RPE in the morning (08:00) and late afternoon (18:00) for the continuous and alternated protocols (CP and AP). Statistical significance (P < 0.05) is indicated in bold.

Variables	Morning CP AP		Late afternoon CP AP		Significance of main effects for time of day	Significance of main effects for protocol	Significance of main effects for TD x Protocol
Tlim (s)	272 ±59	312 ±82	334 ± 57*	380 ±54*	**P < 0.001**	**P = 0.02**	**-**
HRbaseline (bpm)	66.8 ±4.5	67 ±5.4	71.9 ±5.3*	73.1 ±5.5*	**P < 0.001**	**-**	**-**
HRpeak (bpm)	178 ±9	180 ±9	186 ±8*	186 ±8*	**P < 0.001**	**-**	**-**
[La]baseline (mmol/l)	1.9 ±1.9	1.9 ±0.5	1.9 ±0.5	2 ±0.7	**-**	**-**	**-**
[La]peak (mmol/l)	13.9 ±1.9	14.7 ±2.1	16.2 ±2.1*	17.9 ±1.8*	**P < 0.01**	**P < 0.01**	**-**
RPE	17.4 ±0.9	17 ±1.4	16.9 ±1.1	17.2 ±1.4	**-**	**-**	**-**

*: Significantly different from morning value, p < 0.05

-: p > 0.05

### Time to voluntary exhaustion

There was a significant “main effect” of TD on Tlim with lower values in the AM condition compared to the PM condition by 62 s for CP and 68.4 s for AP [F_(1,10)_ = 28.78, P<0.001, η_p_^2^ = 0.74]. The analysis showed a significant “main effect” of protocol on Tlim with lower values in the CP condition compared to the AP condition by 39.1 s at AM and 46.1 s at PM [F(_1,10)_ = 6.88, P = 0.02, η_p_^2^ = 0.4]. No significant (TD × protocol) interaction was obtained for Tlim [F = 0.07, P = 0.78, η_p_^2^ = 0.007].

### Heart rate

There was a significant “main effect” of TD on HRbaseline [F_(1,10)_ = 43.85, P<0.001, η_p_^2^ = 0.81] with higher values at PM compared to AM (P < 0.01). There was a significant “main effect” of TD on HRpeak with lower values in the AM condition compared to the PM condition by 7.4 beats/min for CP and 6.6 beats/min for AP [F_(1,10)_ = 39.07, P<0.001, η_p_^2^ = 0.79]. No significant (TD x protocol) interaction was obtained respectively for HRbaseline nor HRpeak (P = 0.51; P = 0.74).

### Lactate

There was a significant “main effect” of TD on [La]peak with lower values in the AM condition compared to the PM condition by 2.2 mmol/l for CP and 3.2 mmol/l for AP [F_(1,10)_ = 19.4, P<0.01, η_p_^2^ = 0.65]. The analysis showed a significant “main effect” of protocol on [La]peak with lower values in the CP condition compared to the AP condition by 0.7 mmol/l at AM and 1.7 mmol/l at PM [F_(1,10)_ = 12.05, P<0.01, η_p_^2^ = 0.54]. Analysis showed that [La]peak was higher in AP compared to CP in the PM but this difference did not reach significance (P = 0.09). No significant (TD x protocol) interaction was obtained respectively for [La]baseline nor [La]peak (P = 0.85; P = 0.33).

### Rating of perceived exertion

There was no significant difference in RPE values between conditions (P > 0.05), indicating that RPE was similar across all conditions. No significant (TD × protocol) interaction was obtained for RPE (P > 0.05).

## Discussion

The main purpose of this study was to explore the effect of TD on Tlim and physiological responses to AP and CP. The results of the present study indicated that the best performance in AP and CP occurred in the PM with the higher values for HRbaseline, HRpeak and [La]peak compared to AM.

The current finding indicated that Tlim of high-intensity cycling exercise was greater in the PM compared to the AM in both protocols. The results of the present study are in agreement with the results of Hill et al. (1996), which indicated that Tlim was 9% greater (p < 0.01) in the PM (214 +/- 43 sec) than in the AM (196 +/- 38 sec) [20]. Compared to the study of Hill et al. 1996, the exercise intensity in the present study is lower. Since decreasing power output increases aerobic contribution, the Tlim was 18% higher (p = 0.02) in the PM (334 +/- 57 sec) than in the AM (272 +/- 59 sec) for CP and 17% higher (p = 0.01) in the PM (380 +/- 54 sec) than in the AM (312 +/- 82 sec) for AP. The fluctuation observed in Tlim in the two protocols might be explained by the diurnal variation in the aerobic contribution during the exercise.

Moreover, it has been reported that exercise might be maintained for longer in the PM than in the AM, because there is a better work tolerance at high intensity exercise in the PM [22]. In the same context, Hill et al. (1992) [21] showed that the greater amount of work in the PM during exhaustive high-intensity cycling exercise was associated with a 5.1% higher aerobic power and a 5.6% larger anaerobic contribution. Moreover, the aerobic system responded 6% faster in the PM than in the AM in exhaustive high-intensity exercise [20].

The results of the present study did not show a significant difference in Tlim between protocols. However, Tlim was 14% and 13% higher in AP compared to CP, respectively in the AM and PM. We highlight that all participants stopped cycling at 105% APP in AP. The preferred intensity for the participants was 75% APP. We hypothesized that participants were more motivated in AP to complete 10s at 105% APP to cycle at the lower intensity (75% APP) because the idea of “the following 10s will be easier” might give them motivation to finish the 10s at 105% APP. In fact, the engagement in continuous exercise is perceived to be less enjoyable than the engagement in intermittent exercise [42]. Intermittent exercise or AP could therefore be an effective strategy to increase long-term exercise participation and improve human health. The results of the present study might confirm that AP could induce an important increase in [La] compared to CP [38] due to the reduction of mechanical efficiency during intermittent exercise [43]. On the other hand, the present study showed that there is no significant (TD × protocol) interaction for HRpeak and [La]peak in all conditions. The effect of the TD on HR and [La] responses to the exercise were not affected by protocols, which might explain the lack of difference of the TD effects on the Tlim between the two protocols.

The present study is in agreement with the findings of Reilly and Baxter (1983) [22] who reported longer work-times, and consequently higher [La]peak when set bouts of high intensity cycling exercise was performed in the PM compared to the AM. However, Sekir et al 2002 [44] reported that [La] is not influenced by TD when performing a maximal incremental test (increases of 30 W every 2 min). Moreover, Fernandes et al. (2014) [26] found that the [La] responses to 1000-m cycling time-trial were similar for the AM and PM. This contradiction might be due to the daily variations in physical (e.g. muscle) performance [45] and motivation of the participants and/or the difference in methodology (protocol, intensity and duration).

The results of the present study indicate that the TD has no effect on RPE scores. Although these results are in concordance with those of Deschenes et al. (1998) [46], Dalton et al. (1997) [47] and Morris et al. (2009) [48], they are still at odds with those of Faria and Drummond (1982) [16] and Ilmarinen et al. (1980) [49], which showed a diurnal variation in RPE during high intensity exercise with the maximal RPE scores observed in the PM. The disparity between these results seems to be in relation with the psychological status of the subjects and the situation in which the exercise is performed. In fact, the RPE may be influenced by sociological, psychological, environmental factors, and specific characteristics of the subjects [50]. The RPE may be influenced by other factors than the metabolic rate and could be a poor indicator of the intensity of effort during physical activities [51]. It is also important to note that RPE was recorded only once which may be a limitation of the study.

Circadian variation in some cardiovascular parameters such as HR response to exercise is not well explored. The results of the present study show that the HR at rest and HRpeak were higher in the PM than in the AM. Indeed, the rhythm in HR could be retained with similar phase and amplitude to those at rest during exercise [52]. It is well established that the resting HR follows a pattern of circadian rhythmicity [15, 53]. However, the TD effect on maximum HR response to exercise was less studied. Cohen and Muehl (1977) [54] measured HR at rest and during cycling exercise at seven times of the solar day with the lowest HR occurring in the AM. This temporal pattern was evident both during and after exercise. Furthermore, Wahlberg and Astrand (1973) [55] exercised 20 male subjects at 3:00 AM and 3:00 PM and at both submaximal and maximal exercise. HR during exercise was consistently lower in the AM. However, Cohen (1980) [15] demonstrated that the HR response to incremental exercise just prior to exhaustion, when exercise intensity is maximal, does not vary with TD. A more recent study has established a non-significant variation in HR response to 1000m from AM (146 +/- 29 bpm) to PM (154 +/- 14 bmp). In fact, the intensity of exercise may be an important determining factor in the observed circadian oscillation [56, 57].

Finally, we highlight that the longer work-times obtained in the PM than the AM in CP and especially in AP could be responsible for the observed increase in HR and [La] at the PM. Like every study, the present work does have some limitations. The level of motivation of the participants, the BT and oxygen uptake was not measured and the RPE was recorded only at the end of the exercise. It is possible that the homogeneity of the subject population (sex and level of training), or the fact that these subjects were amateur soccer players might have contributed to the present results.

## Conclusions

These outcomes bring further support to the literature that high-intensity cycling exercise was affected by time of day. In the present study, we showed for the first time a diurnal variation in the performance of exhaustive alternated-intensity cycling exercise. For time to voluntary exhaustion, no difference between the alternated and continuous protocol was observed irrespective of the exercise being performed in the morning or late afternoon. Interestingly, different findings were observed from our hypothesis: time to exhaustion was 14% and 13% higher in alternated protocol compared to the continuous protocol, respectively in the morning and late afternoon, possibly due to psychological factors. We suggest that the alternated protocol could be a more effective strategy than continuous protocol to increase engagement in physical activity. Contrary to continuous protocol, the alternated protocol is not investigated. We recommend therefore that future studies include the alternated protocol in the research of sports science.
